# Potential uses of silkworm pupae (*Bombyx mori* L.) in food, feed, and other industries: a systematic review

**DOI:** 10.3389/finsc.2024.1445636

**Published:** 2024-09-17

**Authors:** Luis Miguel Rodríguez-Ortiz, Carlos A. Hincapié, Gustavo Adolfo Hincapié-Llanos, Marisol Osorio

**Affiliations:** ^1^ Grupo de Investigaciones Agroindustriales (GRAIN), Escuela de Ingenierías, Universidad Pontificia, Medellín, Colombia; ^2^ Grupo de investigación en Gestión de la Tecnología y la Innovación (GTI), Escuela de Ingenierías, Universidad Pontificia, Medellín, Colombia

**Keywords:** sericulture by-product, defatted pupae, oil, protein, meal, α-linolenic acid

## Abstract

The increasing pressures imposed on ecosystems by the growing needs of the human population are stimulus for research into innovative and unconventional sources of raw materials for different industries. This systematic review was carried out to investigate the available literature on the possible industrial uses of silkworm (*Bombyx mori* L.) pupae, a residue of silk production. The review was conducted using an adapted version of PRISMA. After a screening process, 105 articles were obtained and subjected to a detailed quantitative and qualitative analysis. It was found that in the last decade there has been a significant increase in the number of papers devoted to the study of the potential use of silkworm pupae in different applications, with a significantly higher number in the last three years of the scope of this review, indicating a growing interest in the subject. From the analysis of the information collected, promising uses in human and animal food, such as fish, mammalian, poultry, swine and companion animals, as well as potential uses for the pharmaceutical industry, were identified. The evaluated research identified compounds with antioxidant activity and important contents of unsaturated fatty acids, which are related to beneficial effects on cardiovascular health, diabetes control, reduction of the risk of developing certain types of cancer and inflammatory activity, among other benefits. One of the most relevant findings is that many studies report a significant concentration of α-linolenic acid in silkworm pupae oil, which is attributed with anticancer, anti-inflammatory, antioxidant, anti-obesity and neuroprotective properties, among others.

## 1 Introduction

Humankind faces numerous challenges, including population growth, climate change, food insecurity, and the increasing loss of natural ecosystems and biodiversity. Projected population growth will require greater food diversity and quantity, and increased research to mitigate its effects and future threats (1). In this context, the United Nations introduced the Sustainable Development Goals in 2015, setting an agenda for global cooperation to ensure that future generations can benefit from the natural resources currently at our disposal (2).

Food production involves significant environmental concerns. For example, approximately 70% of water drawn from sources such as aquifers, rivers, and lakes are used for agriculture (3). greenhouse gas (GHG) emissions from the AFOLU sector (Agriculture, forestry and other land use) account for 22% of global emissions (4). Finding alternative food sources to mitigate the effects of climate change represents a major collective challenge that have been addressed. Ideally, these new alternatives should be easy to produce and represent a lower negative impact on the environment. Such is the case of insects (5, 6), and specifically the silkworm, which are becoming the focus of research in last years (7, 8).

Furthermore, the increased exploitation of natural resources, whether renewable or not, the anthropogenic overproduction of greenhouse gases, and the expansion of agricultural frontiers at the expense of forests and jungles have led to a loss of biodiversity, among other environmental issues (9). Additional factors, including production excesses and inefficiencies in waste management, worsen these problems (9). The above-mentioned elements highlight the urgency of finding innovative and sustainable raw materials that can reduce the environmental footprint of different industries, especially the food industry. That’s why researching the possibilities of materials that have been considered waste in the past is important. Traditionally, silkworm pupae (*Bombyx mori* L.), have been considered a waste material in silk production (10), However, in recent years, studies on its use in different applications have begun to appear. The volume of information that has appeared makes it necessary for it to be organized and catalogued, to make it more visible and available.

Then, a systematic review has been performed, and the result reflects the current global status of potential uses and applications of the silkworm pupae (*Bombyx mori* L.), that produces a significant amount of biomass as a byproduct of the silk industry (11). Sericulture is economically vital, particularly in Asian countries, and the silkworm pupae has spurred substantial research aimed to optimize its use (12). Notably, researchers have investigated the role of silkworm pupae in functional foods, leveraging their antioxidant properties and potential health benefits, such as improving blood quality and preventing arteriosclerosis, liver problems, and thrombosis (13, 14). Additionally, a significant amount of research has examined its role as supplements or alternatives to traditional animal feed components, such as fishmeal (15). Other studies have explored their potential in diets for various ruminants, where they may reduce methane emissions without affecting nutrient intake or digestibility (16). An important number of authors have reported the nutritional characteristics and fatty acid composition of *B. Mori* pupae (17–19). Additionally, the findings show that some researches have been made on acceptance of an insect as ingredient, due to its physical and nutritional characteristics, as for its quality, processing methods, acquisition, transformation, and preservation (20).

The following paper presents that review, based on documents indexed in the Scopus and Web of Science (WOS) databases published between 2001 and 2022, that discuss scientific evaluations of the potential uses of the *B. mori* silkworm pupae as a food ingredient for humans, a dietary supplement for animals, and other uses in medicine and industry. The documents underwent a selection and information analysis process using PRISMA methodology guidelines. From the selected documents, quantitative and qualitative data were collected and classified to consolidate and analyze information in a way that the most relevant uses of the pupae can be identified and its potentialities for different sectors understood.

## 2 Methodology

This systematic review specifically focused on research exploring the uses of the silkworm pupae (*B. mori*). The search was performed through a rigorous and replicable process, ensuring minimal bias and error in the searches, by implementing and adapting the guidelines of the PRISMA 2020 methodology (21, 22). This methodology made easier the selection and collection of both quantitative and qualitative data for eligibility and analysis (23).

Initially, preliminary explorations were conducted to define the search equation for systematically retrieving documents indexed in the Scopus and Web of Science (WOS) databases. Searches were performed within the title, abstract, and keywords fields (TITLE-ABS-KEY), limited to papers published from January 1, 2001, to December 31, 2022, and to English, Portuguese, and Spanish languages.

The primary search was conducted using the terms: “*Bombyx mori*” OR “silkworm” in combination with (pupae OR chrysalis) AND (oil OR flour OR meal). The final search equations were:

- Scopus: (TITLE-ABS-KEY (“Bombyx mori” OR “silkworm”) AND TITLE-ABS-KEY (pupae OR chrysalis) AND TITLE-ABS-KEY (oil OR flour OR meal)).
- Web of Science: (((ALL= (Bombyx mori OR silkworm)) AND ALL=(pupae OR chrysalis)) AND ALL=(oil OR flour OR meal)).

At first, 295 documents were retrieved: 141 from the Scopus database and the remaining 154 from the WOS database. From these, 14 reviews, 4 conference papers, 3 proceeding papers, 2 corrections, 2 book chapters, 1 book, 1 conference review, and 1 meeting abstract were excluded, leaving only the research papers, resulting a total amount of 267 papers for further analysis.

Due to duplication, in both databases, 82 papers were excluded, and 25 more since full access to the text was not allowed, after a search conducted through various sources and mechanisms. The remaining documents underwent title and abstract screening, applying the following exclusion criteria to discard those that did not align with the study objectives and to select the most relevant ones:

- Does not contain relevant information about *B. mori* for this research.
- Not related to the use of *B. mori*.
- Research on sericin from silk cocoon husk was carried out.
- The document was not in the selected languages.
- The studied species was not *B. mori.*
- Information from the document was not available.

**Figure 1:**
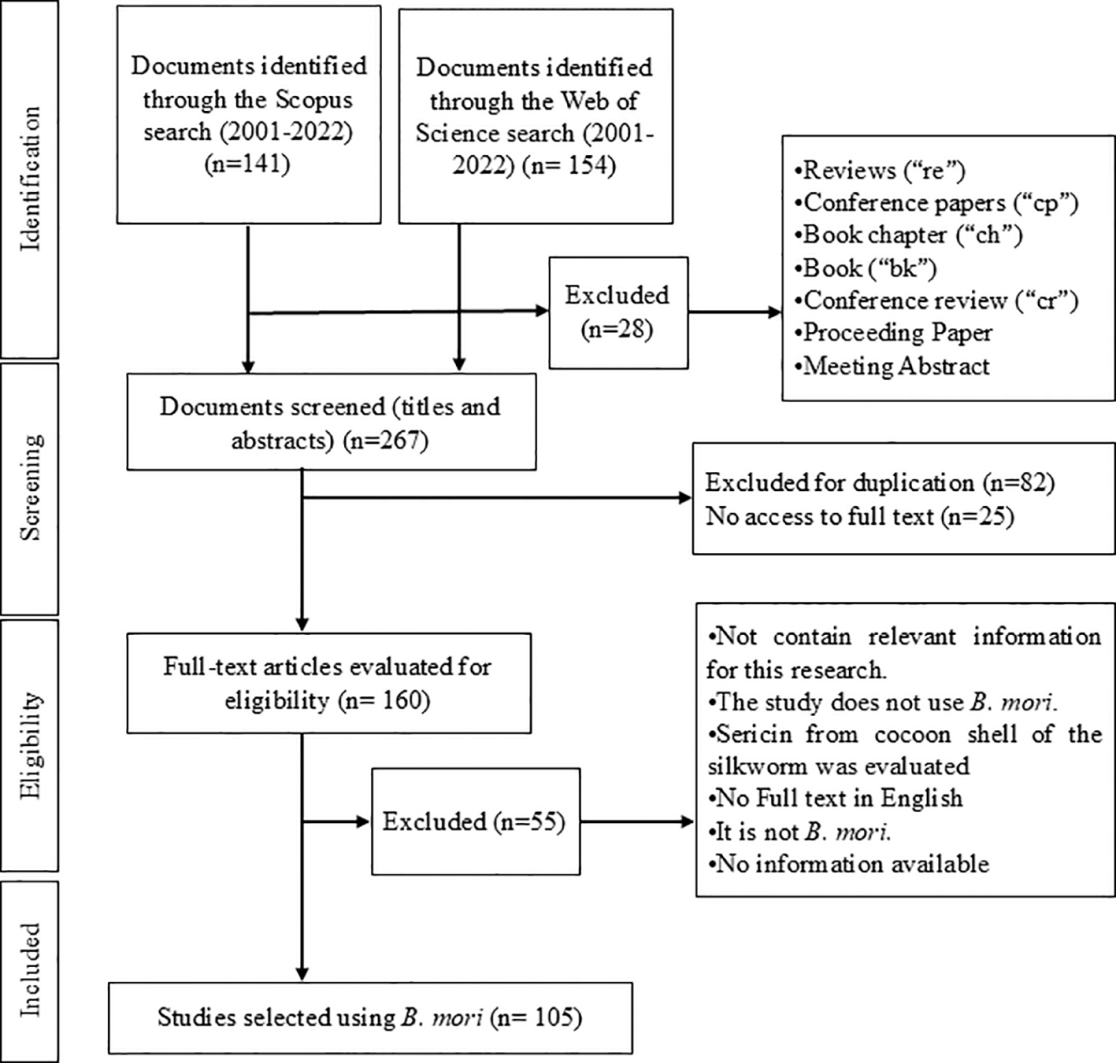
Flowchart inclusion and exclusion documents.

The followed procedure is outlined in **Figure 1**.

The 105 selected documents were thoroughly reviewed to ensure impartiality and the validity of the information, including scrutinizing research funding sources and potential conflicts of interest as prescribed by PRISMA guidelines. Following, a comprehensive reading of each document was performed to identify the most pertinent information, particularly focusing on the use of the silkworm pupae and the degree of transformation it underwent, as well as capturing key findings.

To organize and analyze this information more efficiently, a detailed database was created, containing the fields Authors, Title, Year, Source Title, Affiliations, Country, Keywords, Investigated Compound (Pupae/Oil/Flour/Protein), Specific Use, Use/Applications, and General Use. This database made easier quantitative and qualitative analyses and enabled creation of graphs, maps, tables, and visual representations using VantagePoint software.

The information compiled was used to determine the number of documents generated year by year, resulting in the evolution of research in terms of quantity and topics of interest. From this information, the general trends in terms of potential uses of B. mori pupae that have been researched were established. The most studied by-products were also established and correlated with each of the evaluated uses. After reviewing all the papers, those that performed a proximate analysis were selected and their results were compared with each other. In this way, averages of the composition of the main compounds present in the pupa were obtained. The same was done with the papers that studied the oil obtained from the pupae and the concentrations obtained for the main acids identified were compared. Finally, the main researches were summarized and classified according to the main specific uses identified.

## 3 Results and discussion

**Figure 2:**
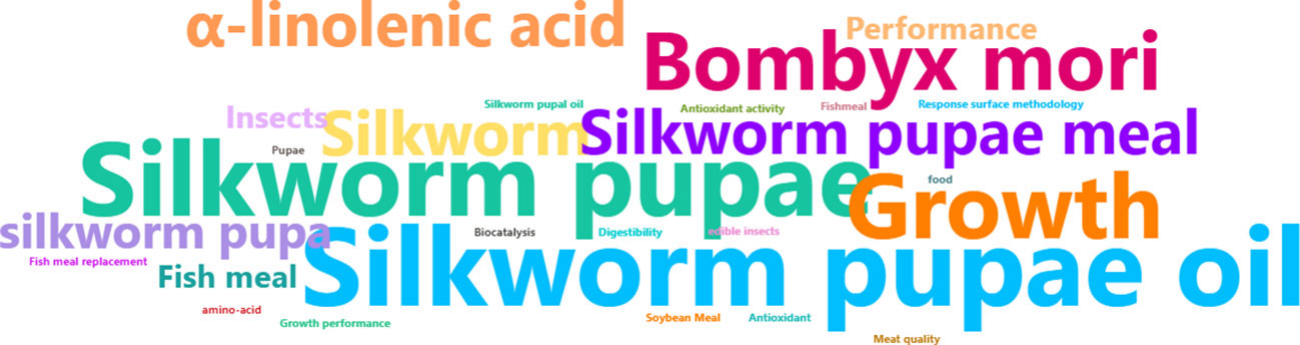
Keyword cloud of reviewed documents.

**Figure 2** was obtained from the results of the linguistic analysis performed using Vantage Point software. The figure displays the keywords that recur more frequently in documents from Scopus and Web of Science, highlighting the most prominent among them, including “Silkworm Pupae oil”, “Silkworm Pupae meal”, “Growth”, “α-linolenic acid”, “Performance”, “Fish meal” and “Insects”. The size of the words in the figure gives an approximate idea of the relative recurrence in the documents. These keywords closely align with the themes thoroughly explored in this review and confirm the appropriateness of the chosen search terms. The size and alignment suggest not only the relevance of the selected keywords but also indicate the topics of highest interest in research.

### 3.1 Trends in publication volume over time

**Figure 3:**
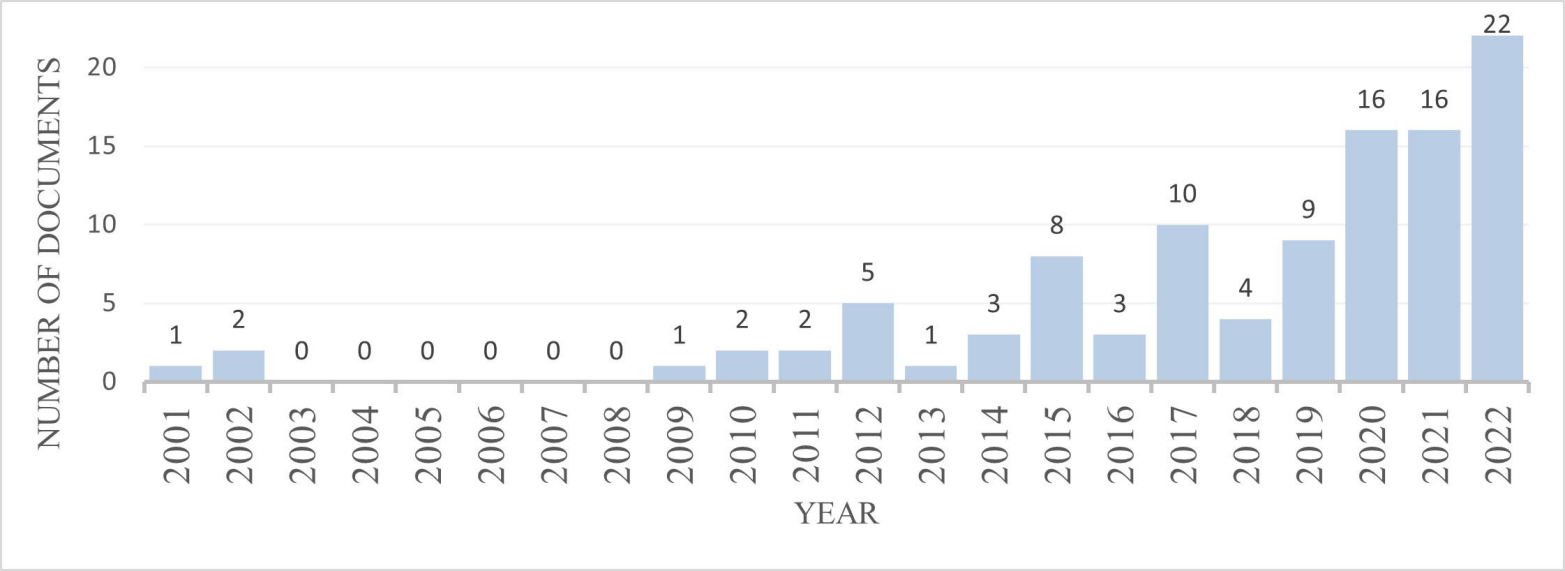
Publications per year.

The evolution of publication numbers over the years was analyzed, showing a rising trend as illustrated in **Figure 3**. In the first 12 years included in the search period, there was minimal article production, with zero to two publications per year. Beginning in 2012, there has been an observable increase in output, with a significantly larger number of studies appearing in the last three years, indicating a growing interest in the topic. The marked surge in publications in 2015 coincides with global milestones related to sustainability concerns, such as the completion of the Millennium Development Goals (24), the significant United Nations Climate Change Conference in Paris and the ratification of the Sustainable Development Goals (2). The coincidence of publication peaks with major international sustainability events suggests that the academic community is responding to global policy changes and the growing need for sustainable solutions in industry. Furthermore, the use of *B. mori* in this area aligns with broader environmental and economic goals, such as reducing dependence on conventional raw materials such as fishmeal, often overexploited and expensive (25). This transition in research focus highlights the adaptability of silkworm as a resource for diverse industries.

### 3.2 Analysis of specific uses of *B. mori* over time

**Figure 4:**
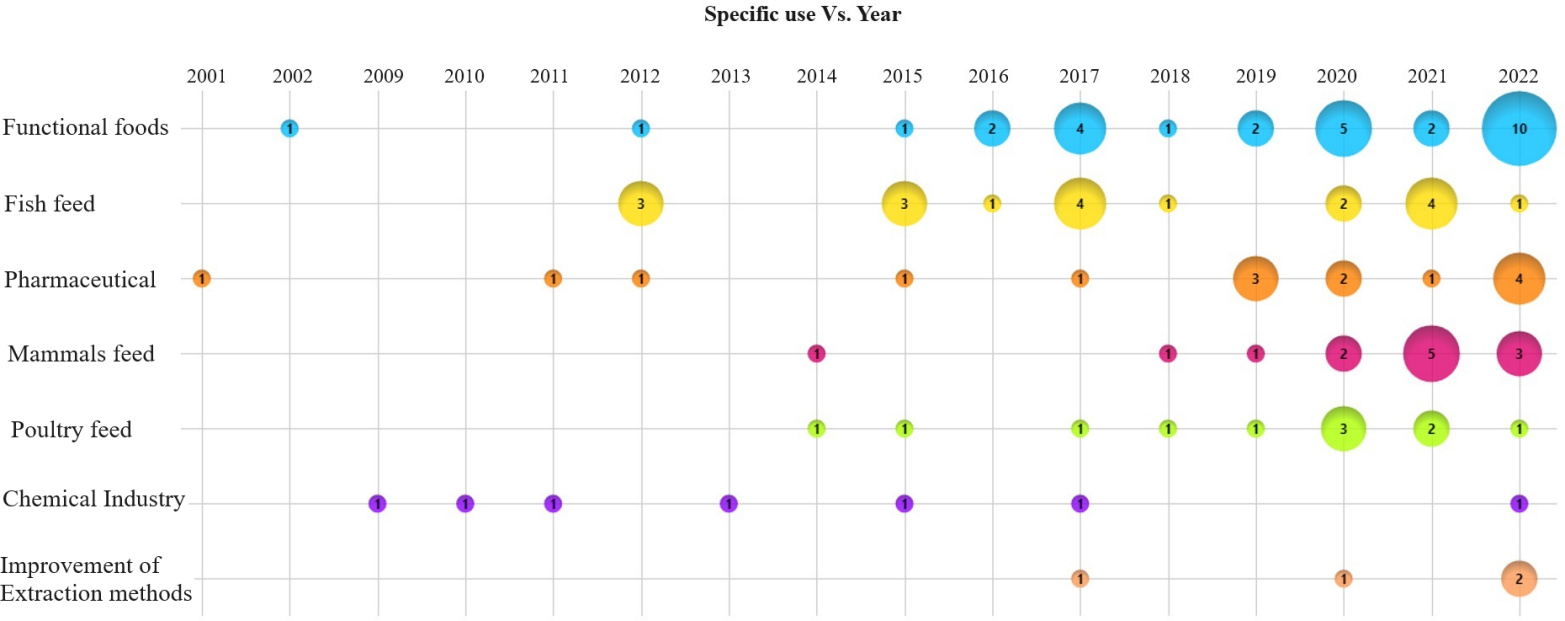
Scientific production: Specific use vs year. The number inside each circle is the quantity of documents that discussed each specific use.

The analysis of specific uses of *B. mori* examined across various documents through the years, as depicted in **Figure 4**, highlights the emergence of different research trends. From 2001 to 2011, research predominantly focused on pharmaceutical applications and uses within the chemical and food industries. Starting in 2012, there was a notable shift toward studies on its use in animal feed, particularly for fish, mollusks and crustaceans (26), poultry (27) and mammals (18).

This shift in research focus probably reflects changes in the priorities of the research community or responses to emerging global needs, such as the search for sustainable sources of animal feed. The trend towards more sustainable and innovative feeding options could be due to increasing pressure on traditional feed sources and the need to reduce the ecological footprint of intensive agriculture, livestock farming and overfishing. The exploration of *B. mori* as an alternative feed source offers potential benefits due to its high protein content, the presence of essential amino acids or health-promoting fatty acids, making it a valuable object of study in the context of sustainable agriculture and aquaculture.

### 3.3 Research trends in the use of *B. Mori* for human and animal nutrition

Recent research on *B. mori* has shown growth in studies related to human nutrition and the pharmaceutical industry, particularly due to its lipid content with functional properties (28, 29) and its richness in unsaturated fatty acids, and antioxidants (30). There has also been a slight increase in publications related to the exploration of techniques for extracting bioactive compounds from the pupae (13).

Additionally, data analyzed using VantagePoint software highlights countries leading in publication volume. In Asia, China, India, and Thailand are prominent sources with 36, 16, and 10 published documents, respectively, followed by South Korea and Japan with 7 publications each. Collectively, these five countries accounted for 62.81% of all analyzed papers.

Research conducted in India and China emphasizes the importance of making the most of biodegradable waste produced by silk factories, where improper disposal of *B. mori* residues can lead to environmental issues including undesirable smells and the spread of dangerous diseases (31). However, these residues, when properly managed, can be effectively used in practical applications such as fuel production (32, 33). In China, Pakistan, and Thailand, studies focus on the use of *B. mori* for animal feed, providing high-quality nutrients to animals without any side physiological effects (34–36). In South Korea and Japan, the insect has been evaluated for human consumption to meet the protein needs of large populations with a smaller carbon footprint (37, 38).

Italy and Poland are next in research production with six documents each, positioning Europe as the second highest source of total publications. In these countries, similar to those mentioned previously, most studies report the primary use of *B. mori* as a supplement and/or substitute for animal nutrition (39–41). This is seen as a potential response to the high costs of traditional concentrates, scarcity of raw materials, and increasing regulations and bans on the use of most meat and bone meals in animal feed (42, 43).

In the Americas, publication volume is lower, but it is led by the United States with 4 publications focused on evaluating and researching the use in animal feed and human nutrition and health (17, 44–46). In Latin America, Brazil stands out, despite of having only 2 publications, which evaluate the addition of the insect in animal diets (18) and as an optimization of the culture medium for the bacterium *Bacillus thuringiensis israelensis*, used in agriculture as a biological pest control agent (47).

### 3.4 Correlation between uses and by-products of *B. mori*

For this analysis, two types of groupings were proposed for the different studies, as shown in **Table 1**. The first grouping is the general use (animal feed, industry, human food, human health, veterinary), and the second is the type of by-product derived from the pupae (pupae when not otherwise specified or untransformed, meal, defatted meal, oil, protein). These groups can be crossed, as shown in **Table 1**, make it easier to visualize the relative number of papers devoted to each topic. Among the by-products, *B. mori* pupae meal stands out with a total of 47 mentions (see **Table 1**). 31 of these references are related to animal feed. In these articles, the meal is proposed as a potential alternative or supplement to traditional feed diets, which in some cases helps minimize feed production costs and environmental pollution (48). Positive results were also reported in the feed conversion efficiency in species such as pigs (49), broiler chickens (50), rabbits (41) and fish (51).

**Table 1:** Number of papers by general use and by type of by-product.

General use	Type of by-product					
	Defatted Meal	Meal	Oil	Protein	Pupae	Total General Use
**Animal Feed**	6	31	4	–	6	47
**Other Industries**	–	3	15	3	2	23
**Human Food**	–	6	7	2	5	20
**Human health**	–	3	13	–	3	19
**Veterinary**	1	4	3	–	2	10
**Total**	7	47	42	5	18	119

Oil was the second most evaluated type of by-product derived from the pupae, with 42 mentions. Of these, 36% of the documents (15 articles) assessed the potential uses of the oil as it is a good source of fatty acids and functional pigments (52). It has been determined that these qualities of the oil make it potentially usable in the food and pharmaceutical industries (13, 30), as well as in the cosmetic (53), and chemical industries (31, 54). Studies examining the composition of the oils concluded that it varies depending on the variety and origin of the pupae (55).

The third most used by-product is the whole pupae itself. Of the 18 mentions, eight focused on animal nutrition and health, analyzing nutritional attributes, ease of cultivation, and biomass production (18, 56). These studies suggest that the silkworm pupae can partially replace fish meal or soybean meal in diets for animals such as poultry (27), fish (19) and rabbits (43), among others. Often, replacement rates of 25% to 30% of the total diet formulation are reported, although some studies indicate that higher rates are possible (19, 35). However, it is crucial to consider that the replacement rate varies depending on the animal species. For example, in rabbit diets, Gugołek et al. (43) report gastrointestinal physiological changes when replacing soybean meal with inclusion rates of 10%, thus recommending not to exceed a 5% replacement rate.

Protein is the least reported type of by-product derived from *B. mori* pupae, with only five publications identified. These studies are associated with the textile industry for fabric dyeing (10), human food evaluated in meat and dairy products (37, 57) and pharmaceutical and veterinary products (58, 59). Notably, these references are mostly from the past five years, with 60% of them appearing in 2022, suggesting that the evaluation of silkworm pupae protein is a relatively new area of research that may present a fruitful avenue for future studies.

### 3.5 Physicochemical analysis of the pupae

Various physicochemical analyses of the pupae have been reported in the literature, particularly highlighting analyses of the dehydrated material with moisture contents below 5%. From the **Table 2**, it is evident that the most significant macromolecules in the dry fraction are protein and ether extract (fat), averaging 57.50% and 24.08%, respectively. Due to the macrocomposition of *B. mori* pupae, research has primarily focused on oil and protein. When delving into more detail, such as the composition of fatty acids (**Table 3**) or antioxidant activity, studies are more directed towards their potential use in functional foods (46, 53, 60–62).

**Table 2:** Summary of Proximate analysis.

	Proximate composition in dry basis (%)					
Reference	Crude Protein	Ethereal Extract	Neutral detergent fiber	Fiber in acid detergent	Carbs	Ash
(18)	53.82	30.73	3.26	1.64	ND	ND
(56)	60.70	25.70	ND	ND	ND	ND
(19)	66.24	20.92	ND	ND	ND	12.84
(17)	53.76	8.09	6.36	6.36	ND	6.36
(35)	49.00	33.84	ND	ND	4.62	3.07
(14)	61.50	25.20	ND	ND	8.20	3.20
**Mean ± SD**	57.50 ± 6.36	24.08 ± 9.04	4.81 ± 2.19	4.00 ± 3.34	6.41 ± 2.53	6.37 ± 4.58

SD, Standard deviation; ND, No data.

The most extensively analyzed component was the oil extracted from *B. mori* pupae, to the point that it is of interest to organize the findings reported in the literature in a separate table, the **Table 3**, that displays the composition of some important fatty acids identified in the oil. Notably, among the saturated fatty acids, palmitic acid stands out with an average value of 23.61%; among the monounsaturated fatty acids, oleic acid at 31.45%; and among the polyunsaturated, α-linolenic acid (ALA) has the highest percentage at 29.90%. From the data gathered, there is a consensus that approximately two-thirds of the oil from *B. mori* consists of unsaturated fatty acids. An interesting finding from the research conducted by Kotake et al. (52), indicates that the content and composition of fatty acids within the oil do not significantly differ with respect to the sex of *B. mori*. This uniformity suggests that the fatty acid profile is stable across different biological variables, which could be beneficial for consistent production and use in various applications.

**Table 3:** Fatty acid composition.

Type of fatty acid	Fatty acid Composition (%)					Mean ± SD
	(63)	(64)	(65)	(66)	(67)	
**Saturated fatty acids**	**28.88**	**31.34**	**28.98**	**30.27**	**24.35**	**28.76 ± 1.17**
**Myristic acid (C14:0)**	ND	0.17	0.18	1.52	ND	0.62 ± 0.78
**Palmitic acid (C16:0)**	22.04	24.16	24.45	27.77	19.62	23.61 ± 2.37
**Stearic acid (C18:0)**	6.84	7.01	4.35	0.98	4.73	4.78 ± 2.82
**Monounsaturated fatty acids**	**34.83**	**38.83**	**32.73**	**31.25**	**31.11**	**33.75 ± 3.29**
**Palmitoleic acid (C16:1)**	0.92	1.29	1.56	6.34	1.39	2.30 ± 2.56
**Oleic acid (C18:1)**	33.91	37.54	31.17	24.91	29.72	31.45 ± 5.33
**Polyunsaturated fatty acids**	**36.29**	**29.55**	**37.32**	**29.61**	**44.03**	**35.36 ± 4.19**
**Linoleic acid (C18:2)**	5.48	4.54	4.64	4.70	7.96	5.46 ± 0.43
**α- Linolenic acid (C18:3)**	30.81	25.01	32.68	24.91	36.07	29.90 ± 3.99

SD, Standard deviation; ND, No data. Bold values are the totals for each fatty acid subset (saturated, monounsaturated, and polyunsaturated).

The rich content of unsaturated fatty acids, particularly oleic acid and ALA, positions *B. mori* oil as a potentially valuable ingredient in the nutraceutical and functional food sectors. ALA has been identified as having anticancer, anti-inflammatory, antioxidant, anti-obesity, neuroprotective and intestinal flora regulating properties, among others (68). Its composition aligns well with current health trends that favor oils with high unsaturated fatty acid content for their beneficial effects on cardiovascular health and overall wellness (69). In addition to cardiovascular benefits, unsaturated fatty acids may play an important role in the management of type 2 diabetes, reduction in the risk of developing certain types of cancer and in the reduction of inflammatory activity, among other benefits (70).

### 3.6 Analysis by specific use

A significant number of documents focus on specific uses such for human food, pharmaceutical applications, and the feeding of fish, poultry, and pigs. It is particularly noteworthy that there is a document which explores the topic of Greenhouse Gases reduction (71). The main specific uses identified are discussed in more detail below.

#### 3.6.1 Functional food applications

Research has shown that the inclusion of *B. mori* meal can impact the rheological, textural, and sensory properties of food products such as whipping cream and ice cream. An optimal concentration of just over 1% can enhance the consistency and extend the shelf life of ice cream, but higher concentrations may adversely affect the flavor and color (72). Another innovative application involves incorporating *B. mori* meal into chicken bread spread, where concentrations up to 50% have been found to be acceptable to consumers. These modifications improve nutritional content and maintain satisfactory sensory properties and physical characteristics (73).

The studies reviewed show high percentages of some unsaturated fatty acids in silkworm oil (54, 74), particularly ALA, an essential omega-3 fatty acid (75, 76). This fatty acid, which includes polyunsaturated fatty acids that do not cause allergies (77) and functional pigments like lutein and neoxanthin (52). There is evidence that these two compounds have anticancer (78) and photoprotective (79) activities. These findings suggests that *B. mori* oil could be a sustainable alternative to traditional vegetable oils, offering comparable nutritional benefits (80).

Research has often looked for the compounds responsible for the recorded activities. The compound on which most of this research has focused is ALA. This fatty acid, from silkworm oil, has been used in trials to develop an analogue of human milk fat, showing potential for creating specialized nutritional products (81). Additionally, microcapsules containing ALA ethyl ester from silkworm pupae have been studied for their emulsion properties, indicating significant improvements in antioxidant capacity which could be utilized in functional food and pharmaceutical applications (30).

Many of the activities that have been studied, and that may potentially have use in functional foods or pharmaceuticals, have so far been tested in laboratory animals. Silkworm oil supplementation in the diet of Wistar rats led to decreased body weight and reduced adipose tissue accumulation in the liver, along with significant reductions in plasma triglycerides and glucose levels. These recorded activities are probably due to the concentration of ALA (32.10% of total fatty acids) (28). In mice, an extract from silkworm pupae showed potential to mitigate the effects of alcohol, demonstrating detoxification activity. This extract was also rich in ALA, which may contribute to its health benefits (41.60% of total fatty acids) (82). Treatment with silkworm pupae oil in mice has been shown to reduce inflammation, oxidative damage, and the area of gastric ulcers induced by hydrochloric acid/ethanol, suggesting potential therapeutic applications for gastrointestinal health (66). These findings underscore the multifaceted benefits of *B. mori* derivatives, which extend beyond traditional uses to include significant potential in functional foods and medical applications.

#### 3.6.2 Animal nutrition and veterinary uses

Research in this field accounts for 48% of the total reviewed documents, focusing on nutrition in fish, poultry, mammals, and to a lesser extent, the feeding of mollusks, crustaceans, and other insects. Most of these works are motivated by the need to explore alternative sources of animal nutrition to address the costs, lack of availability, and negative environmental impact of many traditional sources (35, 83). New sustainable food sources make it possible to evaluate the effects of substituting traditional products by partially or totally modifying the diet of the species. (34).

##### 3.6.2.1 Fish, mollusk and crustacean

Due to the high nutritional value of aquatic foods and their accessibility to many vulnerable populations, the FAO, in its new approach on Blue Transformation, proposes the adoption of more sustainable aquaculture practices through the enhancement of productive capacities. The increase in annual per capita consumption plays a critical role in global food security and nutrition (84). The global consumption of aquatic foods (excluding seaweed), reported at over 157 million tons, has grown at an average annual rate of 3% between 1961 and 2019 (85). The impact on the biodiversity of marine ecosystems underscores the need to reduce the use of fishing-derived ingredients such as fishmeal and fish oil for feed formulation (86). These ingredients possess high nutritional quality but are unsustainable (87). These are some of the reasons why, in recent years, research related to the search for new nutritional sources for fish, including *B. mori*, has increased (22).

Chowdhary et al. (88) evaluated different protein sources on fingerlings of Asian Catfish (*Clarias batrachus*). Among these were silkworm pupae, with inclusion percentages of 20.30% (without the addition of soybean meal) and 15.20% (with also 15.20% soybean meal). The highest growth levels in fish were observed in treatments where the highest percentage of silkworm pupae was used, and no soybean meal was added. Notably, the study conducted by Barlaya et al. (19), which assessed the addition of unconventional ingredients (*B. mori*, plankton and larvae meal of green bottle fly *(Lucilia sericata* Meigen*))* in the diet of *Labeo fimbriatus*, found no statistically significant differences in variables such as final length, survival, and food conversion factor compared to fish fed conventional ingredients. In other studies, it is recommended that the replacement of fishmeal by *B. mori* meal should not exceed 50% as it might lead to irregular intestinal structure and reduced proteases and hepatic enzymes in juvenile jumbo carp (*Cyprinus carpio* L.), probably due to oxidative stress (34, 89). However, these same studies found favorable results in terms of growth performance in juveniles. In contrast, in the same fish species, Xu et al. (90) and Zhou et al. (91) replaced fishmeal with fermented and defatted *B. mori* meal, respectively, combined with rapeseed and wheat, and found that there could be an improvement in non-specific immunity and intestinal tract function.

Compared to other insects, it is reported that in one experiment, juvenile mirror carp (*C. carpio* var. Specularis) were fed four isonitrogenous and isolipidic diets that included black soldier fly oil, silkworm pupae oil, yellow mealworm oil, and a mix of the three insect oils, each at a concentration of 25%. It was found that the diet with black soldier fly oil and the oil mix performed significantly better with respect to other diets in terms of growth indicators, intraperitoneal fat lipid metabolism, liver antioxidant capacity, and inflammatory response in the evaluated fish (92).

Several studies have reported the percentage of *B. mori* addition in the concentrate and the substitution of fish meal, soybean oil, oilcake, and rice bran. **Table 4** lists some of these results.

**Table 4:** Evaluation of percentages with silkworm inclusion.

Species	% of *B. mori* inclusion	Addition/Replaced	Remarks	Reference
**Rainbow trout *Oncorhynchus mykiss* Walbaum**	0 - 5 -10 -15	Fish meal with *B. mori* pupae Meal	10% of fishmeal can be replaced with silkworm pupae without any adverse effects on the values of the feed conversion ratio, specific growth rate, weight gain percent, condition factor, survival rate, protein content, lipid content, or nutrition protein utilization.	(93)
**Jian Carp, *Cyprinus carpio* var. Jian**	0 - 25 - 75 - 100	Soybean oil with *B. mori* chrysalis Oil	Replacing 50% or 70% of soybean oil with *B. mori* oil did not generate negative impacts on fish health and may improve their growth.	(94)
**Carps, *Labeo fimbriatus* & *Cyprinus carpio* Linnaeus.**	0 - 10 - 20 - 30 - 40	Oilcake and rice bran with *B. mori* pupae Meal	Protein and fat digestibility increased with the inclusion of 10% to 30% in *C. carpio*. In *L. fimbriatus*, adding 20% increased protein digestibility, but decreased at levels of 40%.	(95)
**Pacific white shrimp *Litopenaeus vannamei***	0 - 25 - 50 - 75 - 100	Fish meal with *B. mori* Defatted Meal	Completely replacing fishmeal with *B. mori* does not affect shrimp growth and has beneficial effects on diet digestibility, antioxidant capacity, and molting time. However, it is recommended that the substitution level be restricted to a maximum of 75%, as complete replacement may cause diseases in the species.	(25)
**Rainbow shark *Epalzeorhynchos frenatum***	0 - 30 - 40 - 50	Fish meal with *B. mori* pupae meal	With a 50% replacement, positive results can be observed in terms of species growth, and it is concluded that replacing fishmeal up to 30% with B. mori could lead to reductions in the total diet cost.	(96)
**Largemouth bass *Micropterus salmoides***	0 - 10 - 20 - 30 - 40 - 50	Fish meal with fermented *B. mori* pupae meal	Fishmeal can be replaced with *B. mori* in the diet without negative effects on body index performance, food utilization, and growth with substitution values up to 30%.	(97)

In general, various studies have shown that replacing different percentages of diets with *B. mori* meal or oil does not negatively impact the performance of the evaluated species and, in some cases, may even improve their growth (93, 94). Additionally, feeding costs can be reduced by replacing fishmeal by 30% (96), like findings reported by Pada Bag (98) who compared a commercial diet with a diet supplemented with *B. mori* meal.

Factors such as the digestibility of proteins and fats were also evaluated, showing that the appropriate replacement percentages vary by species. (95), recommend replacing 30% of oilcake and rice bran with *B. mori* pupae meal in species such as carps, *L. fimbriatus* and *C. carpio*, with favorable results. Other researchers found that in the Pacific white shrimp (*Litopenaeus vannamei*), replacing more than 75% of fishmeal with *B. mori* meal can have negative effects on animal health (25).

Some physical properties (expansion, hardness, and durability) of the concentrates formulated with *B. mori* meal were analyzed, revealing a decline in water absorption rate, and sinking speed of the pellets. It was also demonstrated that with 15% *B. mori* pupae meal, there was no increase in fat leakage or nutrient leaching in food for gilthead sea bream (*Sparus aurata*) (8).

##### 3.6.2.2 Mammals

Secondly, mammal nutrition and health hold a value of 31% of the total in the general classification. Studies highlight their potential for reducing greenhouse gases produced by ruminants. It is estimated that ruminants generate a total of 5.7 gigatons of CO_2_-eq per year, representing 14.50% of total global emissions (99). A study on sheep found that adding 2% of *B. mori* pupae oil can reduce methane emissions by 15 to 20%, even when its use is intermittent. These results are maintained over the long term and showed weight gain (100).

Regarding nutritional aspects in large ruminants, experiments first carried out *in vitro* (101) and then *in vivo* in cattle, indicated that incorporating up to 30% defatted silkworm pupae meal could replace soybean meal at a lower cost, without affecting nutrient utilization, rumen fermentation patterns, or animal health (102). Based on results obtained in sheep, it would be worthwhile to conduct experiments in cattle to observe effects on methane production.

The addition of *B. mori* pupae or meal in rabbit diets has recorded positive results in terms of body weight improvement. Furthermore, it did not affect the final growth performance of the animals, nor the quality and value of the meat (39). Additionally, it was found that *B. mori* pupae, when used in rabbits, could provide nutrients and active substances such as polyunsaturated fatty acids and antimicrobial peptides. However, it is suggested that the inclusion level should not exceed 4%, as higher percentages have been identified to increase the amount of fat in the muscles of the animals and a significant increase in stomach pH (39, 40).

##### 3.6.2.3 Poultry

For poultry nutrition and health, across a total of 11 studies, researchers have justified the need to explore new alternative sources rich in proteins and essential amino acids that are both economically viable and sustainable. In chickens, researchers found that it was possible to partially replace soybean meal and soybean oil with silkworm meal while achieving satisfactory carcass yield and carcass characteristics with healthy levels of omega-3 fats (103). In another study on broiler chickens, researchers found it possible to replace fish meal with silkworm meal, also obtaining improvements in bird health and reduced feed costs (104). This cost reduction has been corroborated by analyses conducted by Khan (35). Other relevant results from the reviewed articles on poultry can be found in **Table 5**.

**Table 5:** Silkworm in poultry diets.

Species	Remarks	Reference
**Quail**	*B. mori* pupae meal can replace 25% to 75% of fishmeal without affecting the quality or egg production in quails.	(105)
**Cherry Valley ducks (*Anas platyrhynchos domesticus*)**	Ducks were fed with 17 different insect meals, including *B. mori*, showed positive results with no significant variations in terms of health and final quality obtained	(36)
**White Leghorn laying hens**	No significant differences were found in the digestive system of laying hens when soybean was replaced by *B. mori* meal. Moreover, no detrimental effects on growth, egg production, serum biochemistry, or intestinal health of the hens were observed.	(106)
**Turkeys**	The replacement of 10% of soybean meal in turkey diets with insect meal, including *B. mori* and black soldier fly (*Hermetia illucens*), showed positive results in the physiological state of the turkeys with little variation in the final compositions of the animals.	(107)
**Broiler chicken**	Broiler chickens were fed for 42 days with a feed containing *B. mori* meal mixed with other ingredients. Production, performance at slaughter, and sensory quality of the meat after cooking were evaluated. It was found that this feed does not significantly affect daily body weight gain or final body weight compared to the control feed containing traditional ingredients like soybean meal and corn.	(108)
**Fattening quails**	It was identified that a 12.50% supplement of *B. mori* pupae meal can negatively affect nutrient digestibility due to the presence of the biocompound chitin and 1-DNJ	(109)

It is highlighted that the results obtained in the selected articles in this review are mostly positive, and it is evident that replacing up to 75% of ingredients such as soybean meal or fish meal with *B. mori* meal in feed did not generate adverse effects on the profile of birds, egg production, or blood composition (83, 103, 105, 106). However, the study conducted by Dalle et al. (109), reported that the presence of compounds such as chitin and 1-Deoxynojirimycin (1-DNJ) could be detrimental to growing quails, highlighting the need for further research.

##### 3.6.2.4 Pet foods

Some papers classified in this section mention the use of *B. mori* for feeding species such as dogs, rodents, lizards, rabbits, geckos, and generally in the feeding of captive species, especially in zoos, laboratories, and homes (17). The silkworm has been a suitable alternative as a pet food due to its easy handling, which facilitates maintenance and biological safety (110–112). In recent years, there has been growing interest in finding substitutes or supplements in the diets of species such dogs. A study conducted to evaluate the possibility of substituting poultry meal in canine diets with *B. mori* have shown that short periods of feeding and substitution did not present adverse effects on nutrient intake and digestibility, animal weight, fat cover levels in relation to musculature, hematology, blood parameters, and fecal production and moisture, among others (113). Nonetheless, the evaluation of these and more parameters over long substitution periods is essential.

#### 3.6.3 Entomoculture

The use of *B. mori* in entomoculture, an industry dedicated to breeding insects as raw material for other industries, has shown a steadily growing interest in response to the progressive scarcity of food resources (114). For example, the study presented by El-Dakar et al. (115) used the silkworm as a feeding substrate for black soldier fly larvae, which allowed for a higher yield of fatty acids and the possibility of feeding this insect with 100% *B. mori* meal.

#### 3.6.4 Other uses

The resources obtainable in the production and transformation of the silkworm are not fully utilized (61). As observed thus far, a high percentage of the evaluated research has focused on its potential use as food for humans and animal species. However, due to its versatility, composition, and other properties, this review also identified research exploring its potential uses for different industries.

The antioxidant properties found in the insect have spurred the evaluation of pupae protein concentrate for the development of anti-aging cosmetic products (58) and for the elimination of shallow wrinkles or whitening (53). Its synergy with drugs for vascular disorders has also been investigated (116). In one experiment, researchers extracted a protein from the silkworm pupae, which was reinforced with hydroxyapatite. This mixture produced a biopolymer film with good tensile properties and without generating cytotoxicity, making it a potentially safe component for biomedical applications (59).

The *B. mori* pupae has been evaluated as a substrate to produce *Cordyceps militaris* (117) and *Cordyceps cicadae* (118), fungi with functional properties and potential medicinal applications (119), although the results were not as positive compared to those obtained with other types of insects (120).

A theoretical (33) and an experimental (32) evaluation raised the potential of silkworm pupae oil as a biofuel. A novel surfactant was synthesized using *B. mori* pupae oil and pupae protein hydrolysates (31).

In agriculture, the limited availability of vital resources for plant growth, such as phosphorus (P), makes it necessary the search for recovery technologies with new sources and resources to address the threat of potential nutrient scarcity (121). The research conducted by Zhang et al. (122), evaluated different co-fermentation systems to improve the recovery of phosphorus from sludges with Fe-bound P compounds (FePs). Compared to the control, the recovered soluble orthophosphate significantly increased when the co-fermentation system with silkworm chrysalis meal was used.

It must also be noted that some studies have identified risks or precautions that must be taken when using *B. mori* pupae or their components. The oil contains components such as sn-1,3 triacylglycerols including palmitic acid, which when consumed directly can lead to health issues in humans (123). It was identified that a 12.50% supplement of *B. mori* pupae meal can negatively affect the digestibility of nutrients in fattening quails due to the presence of chitin and 1-DNJ (109). In some regions of the world, bioaccumulation of selenium in individuals of *B. mori* has been reported, reaching levels that may pose risks to the health of some animal species (115).

## 4 Conclusions

In the last decade, there has been an observable increase in the number of papers devoted to the study of the possibilities of using the *B. Mori* pupae in different applications, with a significantly larger number of studies appearing in the last three years of the scope of this review, indicating a growing interest in the topic.

With increasing production, which peaked post-2020, generating more than 50% of the total papers taken in account in this review, Asian countries such as China, India, and Thailand lead in document generation, contributing 62.81% of the total.

The systematic review highlights the silkworm pupae as a product with increasing global applications. It is now of interest in various fields such as animal and human nutrition, medicine and veterinary science, and the chemical and pharmaceutical industries, among others.

The performed systematic review shows that the most active research focuses on human and animal nutrition. Typically, the potential for total or partial substitution of traditional raw materials was assessed.

In human nutrition, studies focused on identifying nutritional contents that are useful for preventing and treating gastrointestinal diseases, cardiovascular disorders, hyperglycemia, and hyperlipidemia, among others.

Meal and pupae were the most researched parts| in studies related to human nutrition. Meal, defatted meal, and pupae have primarily been investigated for animal nutrition. Meal, oil, and protein have also been evaluated for their applications in industry and human health.

The compound that received the most interest in a considerable number of publications was α-linolenic acid, which has a substantial concentration in the fatty acids of *B. mori* oil and possesses beneficial properties for human and animal health.

Although it has not been the objective of this work, one possibility for future reviews could be to expand the scope to identify the commercial products in which *B. mori* pupae are already used, and to identify the advantages and disadvantages of its use, compared to traditionally used raw materials.

## Data Availability

Publicly available datasets were analyzed in this study. This data can be found here: https://www.dropbox.com/scl/fo/ethin09agcju7tbgx8ivs/AHobIpjEk2T0rVdpDTCHv0I?rlkey=kr4tf7gnwd4x19yipql1oy0uu&dl=0.
